# Economic Evaluation of Community Water Fluoridation in an Upper-Middle-Income Country

**DOI:** 10.1590/0103-644020245994

**Published:** 2024-12-06

**Authors:** Lorrayne Belotti, Paulo Frazão

**Affiliations:** 1 Department of Politics, Management and Health, School of Public Health, University of São Paulo, Sao Paulo (Sao PauloState), Brazil; 2 Hospital Israelita Albert Einstein - Albert Einstein Center for Studies, Research, and Practices in Primary Health Care and Networks, Sao Paulo, Brazil.

**Keywords:** fluoridation, water supply, dental public health, health economics.

## Abstract

The aim was to estimate the cost-effectiveness and cost-benefit of CWF for schoolchildren according to different population sizes served in Brazil. The economic evaluation was conducted from a societal perspective. Total costs were estimated using four variables across different population sizes: capital cost of initial installation, chemical product costs, system operational costs, and monitoring costs. The effect of CWF was analyzed in the context of the widespread use of fluoridated toothpaste, based on studies with Brazilian population groups. The total cost of dental treatment was estimated, including both direct and indirect costs, with a discount rate of 3.5%. A one-way sensitivity analysis was conducted to test the robustness of the results based on measured parameter values. The costs averted due to CWF were US$174.40 and US$85.67 for children aged 5-8 and 3-12 years, respectively, and US$46.66 for those aged 7-12 years, according to the average effectiveness of CWF for each age group. The cost-effectiveness and cost-benefit ratio results were favorable in all scenarios where the population served was 6,000 or more inhabitants. Scenarios unfavorable to CWF were observed only in populations of up to 2,000 inhabitants. The economic evaluation of CWF in an upper-middle-income country proved to be a cost-effective oral health intervention and more economically advantageous, especially in larger areas for both deciduous and permanent dentition.

## Resumo

O objetivo foi estimar o custo-benefício e custo-efetividade da fluoretação da água de abastecimento público (CWF) para escolares de acordo com diferentes tamanhos populacionais no Brasil. A avaliação econômica foi realizada sob uma perspectiva societal. Os custos totais foram estimados por quatro variáveis em diferentes tamanhos populacionais: custo de capital da instalação inicial, custos de produtos químicos, custos operacionais do sistema e custos de monitoramento. O efeito da CWF foi analisado no contexto do amplo uso de pasta de dente com flúor, com base em estudos com grupos populacionais brasileiros. O custo total do tratamento odontológico foi estimado incluindo custos diretos e indiretos com uma taxa de desconto de 3,5%. Foi conduzida uma análise de sensibilidade unidirecional para testar a robustez dos resultados de acordo com os valores dos parâmetros medidos. Os custos evitados resultantes da CWF foram de US$174,40 e US$85,67 para crianças de 5 a 8 e 3 a 12 anos, respectivamente, e US$46,66 para aquelas de 7 a 12 anos, de acordo com a efetividade média da CWF para cada faixa etária. Os resultados da relação custo-efetividade e custo-benefício foram favoráveis em todos os cenários em que o tamanho da população atendida era de 6.000 ou mais habitantes. Cenários desfavoráveis à CWF foram observados apenas em populações de até 2.000 habitantes. A avaliação econômica da CWF em um país de renda média-alta mostrou ser uma intervenção de saúde bucal custo-efetiva e mais economicamente vantajosa principalmente em áreas maiores em ambas as dentições decídua e permanente.

## Introduction

Despite being largely preventable, oral diseases remain the most prevalent non-communicable diseases globally ^1^. The clinical, aesthetic, functional, emotional, and social implications are significant, and the annual economic impact is substantial, with 64% directly attributable to treatment costs, while the remaining 36% relates to productivity losses due to absenteeism from school and work ^2^. Although there has been a reduction in the prevalence and severity of dental caries, particularly in developed regions, it remains the most common global oral disease. Dental health inequalities persist, with lower socio-economic groups being the most affected ^1^. Income inequality ^3^, low human development, and economic indicators, such as annual Gross Domestic Product per capita ^4^, have been linked to its higher prevalence.

In this context, Community Water Fluoridation (CWF) is an important public health strategy for the prevention of dental caries at the population level. Besides its low cost relative to its high social benefit, CWF reduces social inequality in access to fluoride, benefiting all strata of the population served by the public water supply ^5^. A recent Cochrane systematic review reported that water fluoridation is effective in reducing caries levels in both deciduous and permanent dentition in children ^6^.

In Brazil, the implementation of water fluoridation began in 1953, and it became mandatory in water treatment plants in 1974. Between 1970 and 1990, urban water supply coverage increased from 54% to 90%, while fluoridation coverage rose from 3% to 42% ^7^. By 2008, about 144 million Brazilians, or 76.3% of the population, had access to fluoridated water ^8^. The most recent study on fluoridation coverage revealed that, in 2015, 78.6% of the population had access to water adjusted for fluoride at 0.7 mg/L (ppm) in municipalities with 50,000 or more inhabitants ^9^. However, it is important to note that there are differences in the quality of fluoridation among municipalities. A study conducted in São Paulo, Brazil, showed that municipalities with smaller populations, lower per capita income, and not operated by the state sanitation company had lower compliance rates with this measure ^10^.

Despite the high coverage of CWF in Brazil, similar to countries like the USA (63.4%) ^11^ and Australia (88.5%) ^12^, studies that simultaneously analyze its costs, effectiveness, and benefits in contexts of varying population sizes are scarce. A study conducted in both fluoridated and non-fluoridated communities in the USA, with observed caries reductions, concluded that water fluoridation remained cost-saving except in communities with fewer than 5,000 residents ^13^. Similarly, a study from New Zealand showed that CWF remained cost-effective for communities over 5,000 inhabitants under all scenarios when sensitivity analysis was conducted ^14^. A scoping review comprising 24 studies on the economic evaluation of CWF for dental caries prevention, published after 1973, indicated that few studies produced estimates according to community size ^15^.

The economic evaluation of CWF is crucial for advancing scientific knowledge in different sociodemographic and cultural contexts, especially in populations exposed to the widespread use of fluoridated toothpaste ^16^. Moreover, it can inform decision-making by health authorities and managers of public and private companies in the sanitation sector regarding the installation or discontinuation of fluoridation systems in various locations.

Given these aspects, the objective of this study was to estimate the cost-effectiveness and cost-benefit of fluoridation of public water supplies for schoolchildren according to different population sizes served in Brazil.

## Materials and Methods

The cost-effectiveness (CEA) and cost-benefit analyses (CBA) were conducted from a societal perspective, by methodologies applied in previous analyses ^14^ ^,^ ^17^ ^,^ ^18^ ^,^ ^19^. This perspective includes the expenses incurred by the patient and his/her family, either to access the service or other costs that can be considered as a consequence of the intervention such as costs for lost productivity and transportation costs to and from the dental office.

### Costs associated with CWF

CWF cost estimates were based on data reported in a published study that included costs for seven Brazilian population sizes, ranging from less than 2,000 to more than 500,000, between 2012 and 2017^20^. The total costs were estimated by four variables: capital cost of initial installation, chemical product costs, system's operational cost, and costs of monitoring. Within the cost of initial installation, the equipment costs, installation costs (85% of the equipment costs), and costs of technical consultancy (15% of the cost of initial installation) were considered. The operational costs included depreciation and maintenance costs of equipment, which represent about 10% of the initial capital, and the human resource costs of one employee for each water treatment plant, calculated using the average annual cost of wages, plus labor charges. From the available data, annual capital costs of initial installation were calculated at a base discount rate of 3.5% and a base lifespan for capital equipment of 20 years^21^.

### Caries averted

The annual estimates of caries averted were taken from a systematic review conducted regarding the effect of community water fluoridation (CWF) in the context of the wide use of fluoridated toothpaste, based on studies with Brazilian population groups^22^. The evidence was obtained from observational studies with concurrent controls and data for a single point in time comparing the caries index in fluoridated areas to non-fluoridated areas. To control the confounding bias owning to the social differences among the areas, sociodemographic and socioeconomic data from the areas investigated were accessed and the papers were classified according to their comparability. The meta-analysis included ten comparable studies published between 1998 and 2018. The difference in mean dmft between fluoridated and non-fluoridated areas was obtained from children with 5-8 and with 3-12 years-old and the difference in mean DMFT was taken from children with 7-12 years-old. More details can be obtained elsewhere ^22^.

### Costs of treatment for dental caries

The total cost of dental treatment was estimated including direct and indirect costs. Direct costs referred to a complete examination and filling of two dental surfaces, with amalgam, according to the Brazilian Classification of Dental Procedures in 2019 (CBHPO). Indirect costs included transportation expenses and estimated productivity loss of three hours spent with the commute and the dental appointment^14^ ^,^ ^17^, both based on the average hourly wage rate in 2019. These costs were regarding one visit and four public transportation tickets since in all scenarios the patient needed to be accompanied by an adult. The average public transportation fare was US$1.01 in 2019, according to the National Urban Mobility Survey^23^ (Table 1).

**Table 1:** Description of fees for treatment in societal perspective.

Description	Quantification	Value	Source
Societal perspective			
*1. Direct Costs*			
a- Complete examination	1	US$ 27.10	CBHPO¹
b- Filling on 2 surfaces, amalgam	1	US$ 41.89	CBHPO¹
*2. Indirect Costs*			
a- Loss of productivity	180 min	US$ 3.45	Decree nº 9661²
b- Transportation	4	US$ 4.06	BRASIL, 2019³

In 2019, US$1 = R$3.95

1.Brazilian Classification of Dental Procedures in 2019 (CBHPO), available in <https://www.soepar.org.br/tabelaReferencial.php/>

2.Decree nº 9661 - Regulates Law No. 13,152, of July 29, 2015, which provides for the value of the minimum wage and its long-term valuation policy. Avaiable in: <http://www.planalto.gov.br/ccivil_03/_ato2019-2022/2019/decreto/D9661.htm>

3.BRASIL. National Urban Mobility Survey, 2019. Avaiable in: <https://www.gov.br/mdr/pt-br/assuntos/mobilidade-e-servicos-urbanos/pesquisa-nacional-de-mobilidade-urbana-2019>

In line with previous studies, the following assumptions were made: (a) cost of adverse side effects (dental fluorosis) were assumed to be negligible and not attributed a value^17^ ^,^ ^24^ ^,^ ^25^; (b) all carious surfaces would be treated and treatment would comprise of a two-surface dental amalgam restoration per dmft/DMFT^14^. The cost of dental treatment was divided by the lifespan of the amalgam restorations equal to 12.8 years^26^. To determine annual cost averted was applied to a discount rate of 3.5%.

The results are presented in United States Dollars (USD) based on the average exchange rate between the Brazilian Real (BRA) and USD between January 1, 2019 and December 31, 2019 (BRA 1 = USD 0.2535)^27^.

### Costs-Effectiveness and cost-benefit of CWF

The cost-effectiveness was estimated by dividing the annual *per capita* net cost of the CWF with the difference in mean dmft/DMFT resulting from CWF^14^. The net cost was calculated by subtracting costs associated with CWF from annual costs averted^13^. The cost-benefit was estimated according to Kroon and Van Wyk (2012)^19^, in which the cost of the implementation of water fluoridation was divided by the annual costs averted. A program should be considered for implementation and maintenance if cost-benefit is <1^18^.

### Sensitivity Analysis

One-way sensitivity analysis was conducted to test the robustness of the results according to measured parameter values^28^. It provided one plausible variety of savings that could be obtained from CWF. Therefore, sensitivity analysis for the societal perspective was conducted on: ^1^ filling on one amalgam-based surface; ^2^ filling on two resin-based surfaces considering the lifespan of 7.8 years^26^; ^3^ upper and lower levels of estimated effectiveness of CWF; ^4^ discount rates at 0% and 7%.

## Results

The mean annual per capita cost of CWF varied according to the population size, ranging from US$ 7.35 for populations with fewer than 2,000 inhabitants to US$ 0.14 for 520,000 inhabitants. Specifically, the cost was US$ 2.05 for 6,000 inhabitants, US$ 1.45 for 9,000, US$ 0.44 for 30,000, US$ 0.42 for 70,000, and US$ 0.26 for 160,000 inhabitants and US$ 0.26 and US$ 0.14 for 160 and 520 thousand inhabitants, respectively (Table 2).

**Table 2.** Annual CWF costs *per capita*, from 2012 to 2017 (base discount rate of 3.5% and a base lifespan for capital equipment of 20 years).

Size of population served	**Mean Annual *per capita* of CWF with 95% CI**
Small 1 (< 2000)	US$ 7.35 (6.62, 8.08)
Small 2 (6000)	US$ 2.05 (1.82, 2.28)
Small 3 (9000)	US$ 1.45 (1.29, 1.61)
Medium 1 (30000)	US$ 0.44 (0.38, 0.49)
Medium 2 (70000)	US$ 0.42 (0.37, 0.48)
Large 1 (160000)	US$ 0.26 (0.19, 0.33)
Large 2 (520000)	US$ 0.14 (0.12, 0.17)

The mean difference between non-fluoridated and fluoridated areas in the dmft was -2.28 (95%CI -3.26; -1.30) for children aged 5-8 years and -1.12 (95% CI -1.93; -0.32) for children aged 3-12 years. Whereas the difference in the DMFT was -0.61 (95%IC -0.80; -0.42) for children aged 7-12 years (Table 3).

**Table 3:** Difference in mean dmft/DMFT between fluoridated and non-fluoridated areas

Age	Outcomes	Mean difference with 95% CI
5 to 8	dmft	-2.28 (-3.26, -1.30)
3 to 12	dmft	-1.12 (-1.93, -0.32)
7 to 12	DMFT	-0.61 (-0.80, -0.42)

From the societal perspective, the total cost of a two-surface dental amalgam restoration, including direct and indirect costs, was US$76.50. The costs averted resulting from CWF were US$174.40 and US$85.67 for children aged 5-8 and 3-12 years, respectively, and US$46.66 for those aged of 7-12 years, according to the average effectiveness of the CWF for each group age.

Similar to cost-effectiveness, cost-benefit ratio results were favorable in all scenarios where the size of population served was 6,000 or more inhabitants. For example, in the category Small 2 (6 thousand habitants), the ratio value at deciduous dentition was 0.12 in 5-8 years-old schoolchildren. It means that for every 12 dollars spent with CWF provision the per capita saved amount was 100 dollars which means for every dollar spent with the intervention 8,3 dollars were saved. Then, it is licit to conclude that for every dollar spent with CWF provision the per capita saved amount varied at deciduous dentition from US$ 8.3 to 100 in 5-8 years-old schoolchildren and from US$ 4.1 to 50 in 3-12 year’s schoolchildren. At permanent dentition, the values saved varied from US$ 2.2 to 33.3 in 7-12 year’s schoolchildren (Table 4). The result was unfavorable for the CWF in only a scenario: size small 1 for the age from 7 to 12 years old.

**Table 4:** Cost Effectiveness and Cost Benefit by population size (societal perspective).

	Cost-Effectiveness			Cost-Benefit		
Size of population served	dmft		DMFT	dmft		DMFT
	5 to 8	3 to 12	7 to 12	5 to 8	3 to 12	7 to 12
Small 1 (< 2000)	-4.29	-0.95	4.53	0.43	0.87	1.60
Small 2 (6000)	-6.62	-5.69	-4.16	0.12	0.24	0.45
Small 3 (9000)	-6.88	-6.22	-5.14	0.08	0.17	0.32
Medium 1 (30000)	-7.32	-7.12	-6.80	0.03	0.05	0.10
Medium 2 (70000)	-7.33	-7.14	-6.83	0.02	0.05	0.09
Large 1 (160000)	-7.40	-7.28	-7.09	0.02	0.03	0.06
Large 2 (520000)	-7.46	-7.39	-7.29	0.01	0.02	0.03

When the variables were adjusted for sensitivity analysis, cost-effectiveness and cost-benefit remained favorable to CWF for all ages in areas with 6,000 inhabitants or more. The negative variation of cost-effectiveness increased from 3.3 points (-4.2 to -7.5) to 12.0 points (-1.1 to -13.1) according to parameters applied. The main factors were the effectiveness of CWF and the kind of treatment offered. While decreased effectiveness compromised the cost-effectiveness, treatment based on two-surface resin filling became CWF even more cost-effective. The results were similar to cost-benefit. Scenarios unfavorable to CWF were observed only in size up to 2,000 inhabitants.

**Table 5:** Sensitivity Analysis Range for CWF (societal perspective) by population size.

	Cost-Effectiveness			Cost-Benefit		
	dmft		DMFT	dmft		DMFT
	5 to 8	3 to 12	7 to 12	5 to 8	3 to 12	7 to 12
Small 1 (< 2000)						
1- Resin (two faces)	-9.98	-6.64	-1.16	0.24	0.50	0.91
2- Amalgam (one face)	-3.72	-0.38	5.10	0.46	0.94	1.73
3- Lower effectiveness	-1.86	15.45	9.98	0.75	3.06	2.33
4- Higher effectiveness	-5.26	-3.71	1.67	0.30	0.51	1.22
5- Lower discount rate	-2.75	0.59	6.07	0.54	1.10	2.02
6- Higher discount rate	-6.02	-2.68	2.81	0.35	0.71	1.30
Small 2 (6000)						
1- Resin (two faces)	-12.31	-11.38	-9.85	0.07	0.14	0.25
2- Amalgam (one face)	-6.05	-5.12	-3.59	0.13	0.26	0.48
3- Lower effectiveness	-5.94	-1.11	-2.64	0.21	0.85	0.65
4- Higher effectiveness	-6.89	-6.45	-4.95	0.08	0.14	0.34
5- Lower discount rate	-5.08	-4.15	-2.62	0.15	0.31	0.56
6- Higher discount rate	-8.34	-7.41	-5.88	0.10	0.20	0.36
Small 3 (9000)						
1- Resin (two faces)	-12.57	-11.91	-10.83	0.05	0.10	0.18
2- Amalgam (one face)	-6.31	-5.65	-4.57	0.13	0.26	0.48
3- Lower effectiveness	-6.40	-2.99	-4.06	0.15	0.60	0.46
4- Higher effectiveness	-7.07	-6.77	-5.70	0.06	0.10	0.24
5- Lower discount rate	-5.34	-4.68	-3.60	0.11	0.22	0.40
6- Higher discount rate	-8.61	-7.95	-6.86	0.07	0.14	0.26
Medium 1 (30000)						
1- Resin (two faces)	-13.01	-12.81	-12.48	0.01	0.03	0.05
2- Amalgam (one face)	-6.75	-6.55	-6.23	0.03	0.06	0.10
3- Lower effectiveness	-7.18	-6.14	-6.47	0.05	0.18	0.14
4- Higher effectiveness	-7.38	-7.29	-6.97	0.02	0.03	0.07
5- Lower discount rate	-5.78	-5.58	-5.25	0.03	0.07	0.12
6- Higher discount rate	-9.05	-8.85	-8.52	0.02	0.04	0.08
Medium 2 (70000)						
1- Resin (two faces)	-13.02	-12.83	-12.52	0.01	0.03	0.05
2- Amalgam (one face)	-6.76	-6.57	-6.26	0.03	0.05	0.10
3- Lower effectiveness	-7.19	-6.20	-6.52	0.04	0.17	0.13
4- Higher effectiveness	-7.39	-7.30	-6.99	0.02	0.03	0.07
5- Lower discount rate	-5.79	-5.60	-5.29	0.03	0.06	0.12
6- Higher discount rate	-9.06	-8.87	-8.55	0.02	0.04	0.07
Large 1(160000)						
1- Resin (two faces)	-13.09	-12.97	-12.78	0.01	0.02	0.03
2- Amalgam (one face)	-6.83	-6.72	-6.52	0.02	0.03	0.06
3- Lower effectiveness	-7.32	-6.70	-6.90	0.03	0.11	0.08
4- Higher effectiveness	-7.44	-7.38	-7.19	0.01	0.02	0.04
5- Lower discount rate	-5.86	-5.74	-5.55	0.02	0.04	0.07
6- Higher discount rate	-9.13	-9.01	-8.82	0.01	0.03	0.05
Large 2 (520000)						
1- Resin (two faces)	-13.14	-13.08	-12.98	0.00	0.01	0.02
2- Amalgam (one face)	-6.89	-6.82	-6.72	0.01	0.02	0.03
3- Lower effectiveness	-7.41	-7.08	-7.18	0.01	0.06	0.04
4- Higher effectiveness	-7.47	-7.44	-7.34	0.01	0.01	0.02
5- Lower discount rate	-5.91	-5.85	-5.75	0.01	0.02	0.04
6- Higher discount rate	-9.18	-9.12	-9.01	0.01	0.01	0.02

## Discussion

The economic evaluation of CWF in an upper-middle-income country demonstrated it to be a cost-effective oral health intervention. Its economic advantages are particularly pronounced in larger areas and across both deciduous and permanent dentitions. These findings are of significant importance, given the widespread use of fluoridated dentifrice in the study area ^29^. Sensitivity analysis indicated that the effectiveness of CWF and the type of restorative dental treatment are the most critical factors for both cost-effectiveness and cost-benefit analyses.

The annual per capita cost of CWF ranged from US$ 0.14 to US$ 7.35, showing a strong relationship between community size and cost-effectiveness/benefit. A study in Florida, USA, conducted across 44 communities with varying population sizes between 1981 and 1989, found that the cost of fluoridated public water supply was highly dependent on the organizational structure of the supply system and the population size ^30^.

Because annual program costs varied by population size, net savings did as well. Differences in cost-effectiveness according to population size have also been observed in Australia. In communities with fewer than 5,000 inhabitants, the cost-effectiveness for children was -2.23, whereas in those with more than 50,000 inhabitants, it was -6.09 ^14^. In the United States, net savings for communities with 20,000 or more people represented a greater percentage of total net savings than those for smaller populations, due to lower per capita fluoridation costs and a larger number of people served ^31^.

A systematic review conducted in 2014, which included studies from the U.S., Australia, Canada, and New Zealand, reported that the benefit of CWF exceeded costs, indicating a positive rate of return on investment. The review highlighted that CWF saved money from a societal perspective and reduced caries. However, for small communities with fewer than 1,000 inhabitants, the per capita annual cost exceeded the per capita annual benefit ^32^.

Tchouaket et al. (2013) ^33^ analyzed the cost-effectiveness of CWF by simulating various scenarios of reductions in dental caries attributed to the intervention, ranging from 1% to 50%. Despite a different methodological approach, their results aligned with this study, and sensitivity analyses showed that if CWF reduced tooth decay by 1%, one dollar invested in the fluoridation program would save $7.32 to $8.53 in dental care costs per inhabitant in 2010 in Quebec.

The effectiveness levels used in this study were based on adjusted mean differences in dmft/DMFT between fluoridated and non-fluoridated communities. As the effect of CWF was greater in deciduous dentition than in permanent dentition, the cost-effectiveness and cost-benefit ratios were more favorable for deciduous dentition. Nevertheless, the results showed significant savings provided by CWF in permanent dentition. It is worth noting that this advantage for public health intervention could be lower if dental treatment were simpler and based on new approaches, such as minimally invasive dentistry. However, CWF would still be cost-effective, as dental team costs account for most dental treatment expenses ^34^.

Effectiveness and the type of restorative dental treatment were the most influential factors in the sensitivity analyses, particularly in smaller scenarios. In larger populations served, even under worst-case analysis, CWF remained cost-effective. Other studies have similarly found that the cost-effectiveness/benefit of CWF is sensitive to factors such as discount rate, effectiveness of CWF, caries increment, and the type and lifespan of restorations ^14^ ^,^ ^17^.

The model presented in this article enabled cost estimation and confirmed the advantages of CWF, even within the context of widespread use of fluoridated toothpaste. It is important to emphasize that the costs in this study were obtained from a case study involving seven different population sizes in a Brazilian state. However, the cost of a public health intervention can vary across countries and cities, depending on factors such as system design, availability and type of chemicals used, equipment, adjustment of natural fluoride levels, number of fluoride injection points, access to health care, age distribution, and population size.

It is also worth noting that some benefits of CWF could not be included in the adopted model, such as social acceptability due to the retention of teeth, avoidance of extractions and dental implants, and reduced pain and discomfort, leading to less time lost from school or work. Moreover, the savings would be higher in the Midwest, Northeast, and North macro-regions of the country, where oral health data from 2003 and 2010, the two largest and earliest national surveys, showed higher levels of dental caries among 12-year-old children ^34^ and adults ^35^.

One might argue that the costs of treatment for fluorosis were not considered. However, data on fluorosis prevalence have shown low values in the South and Southeast macro-regions, where more municipalities provide CWF and socioeconomic conditions are better ^36^. A significant proportion of fluorosis cases requiring treatment has been observed only in areas with naturally occurring fluoride levels above those recommended for caries prevention ^37^. In addition, confounding factors, such as oral hygiene habits, exposure to other fluoride sources, and diet, were not controlled for.

## Conclusion

In conclusion, CWF represented a negative net cost (cost-saving) in all scenarios, except in populations of up to 2,000 inhabitants for children aged 7 to 12 years. For every dollar spent on CWF provision, the per capita savings varied depending on the type of dentition, age group, and population size. In permanent dentition, the cost savings reached up to US$ 33.3 per schoolchild.

## Data Availability

All data generated or analyzed during this study are included in this article. Further enquiries can be directed to the corresponding author.
